# mHealth interventions to reduce stress in healthcare workers (fitcor): study protocol for a randomized controlled trial

**DOI:** 10.1186/s13063-023-07182-7

**Published:** 2023-03-04

**Authors:** Hannes Baumann, Luis Heuel, Laura L. Bischoff, Bettina Wollesen

**Affiliations:** 1 Institute of Biological Psychology and Neuroergonomics, Technical University of Berlin, Fasanenstr. 1, 10623 Berlin, Germany; 2 Institute of Human Movement Science, University of Hamburg, Turmweg 2, 20148 Hamburg, Germany; 3 Institute of Interdisciplinary Exercise Science and Sports Medicine, Medical School Hamburg, Am Kaiserkai 1, Hamburg Hamburg, 20457 Germany

**Keywords:** Healthcare, Care work, Stress, Stress coping, Digital health technologies, Health intervention, App-based intervention, Digital intervention, eHealth, mHealth

## Abstract

**Background:**

Causes and consequences of chronic stress levels in the context of healthcare work are well examined. Nevertheless, the implementation and evaluation of high-quality interventions to reduce stress of healthcare workers is still missing. Internet and app-based interventions are a promising venue for providing interventions for stress reduction to a population that is otherwise difficult to reach due to shift work and time constraints in general. To do so, we developed the internet and app-based intervention (fitcor), a digital coaching of individual stress coping for health care workers.

**Methods:**

We applied the SPIRIT (Standard Protocol Items: Recommendations for Interventional Trials) statement as a guideline for the present protocol. A randomized controlled trial will be conducted. There are five different intervention groups and one waiting control group. To achieve the sample sizes required by power analysis (G*Power) (β-error 80%; effect size 0.25), the sample sizes of the respective scenarios will be at best as follows: 336 care workers from hospitals, 192 administrative health personnel, 145 care workers from stationary elderly care homes, and 145 care workers from ambulatory care providers in Germany. Participants will randomly be assigned to one of five different intervention groups. A crossover design with a waiting control group is planned. Interventions will be accompanied by three measurement points, first a baseline measure, second a post-intervention measure directly after completion of the intervention, and a follow-up measure 6 weeks after completion of the intervention. At all three measurement points, perceived team conflict, work-related experience patterns, personality, satisfaction with internet-based training, and back pain will be assessed using questionnaires, as well as heart rate variability, sleep quality, and daily movement will be recorded using an advanced sensor.

**Discussion:**

Workers in the health care sector increasingly face high job demands and stress levels. Traditional health interventions fail to reach the respective population due to organizational constraints. Implementation of digital health interventions has been found to improve stress coping behavior; however, the evidence in health care settings has not been established. To the best of our knowledge, fitcor is the first internet and app-based intervention to reduce stress among nursing and administrative health care personnel.

**Trial registration:**

The trial was registered at DRKS.de on 12 July 2021, registration number: DRKS00024605.

## Introduction

### Background and rationale

Occupational psychosocial stress can increase the risk to develop psychological, musculoskeletal, or cardiovascular disease [1, 2]. Especially health personnel around the globe experience exceptionally high levels of occupational stress [3] leading to serious individual, organizational, and societal problems [4]. Additionally, healthcare institutions in various European countries indicate staff shortages, and often fail to retain long-term personnel, exposing health personnel to a vicious cycle of stress and extra work [5]. This is in line with findings in Germany that report job demands in the health sector to be considerably higher compared to other professions [6]. Similarly, within the European Union, it is well known that more than one in four nurses are overburdened [7]. Recurring stressors in health organizations include high work demands, leadership style, few participation opportunities for work structuring, emotional burdens, lack of appreciation, and work-family conflicts [8, 9]. In turn, these stressors may influence recreational activities of affected persons. For instance, sleep and physical activity have been found to be poor in stressed individuals [10, 11]. Additionally, individuals with lower heart rate variability (HRV) were more likely to report poorer sleep quality in the context of chronic stressor exposure than individuals with higher HRV [12]. Frequent or chronic occupational stress is linked to serious health consequences. If work-related demands outweigh individual, social, and organizational resources [13], affected persons may incur psychological and physiological consequences such as sleep disorders, gastrointestinal complaints, burnout, diabetes, and coronary heart disease [14–17]. In severe cases, inability to work can lead to long-term sickness absenteeism [18].

In general, psychosocial stressful stimuli activate neuronal, neuroendocrine, and endocrine pathways. A physiological response to stress occurs thus, among others, at the neurological level, through receptors of the sympathetic nervous system that stimulate the sympatico-adrenomedullary axis. The hormones adrenaline and noradrenaline are released in the adrenocortical medulla, leading to an increase in heart rate and a decrease in heart rate variability (HRV) under physical or psychological stress [19]. Such biological responses to stressful stimuli may be adaptative. However, extreme, frequent, or chronic activations of stress axes may be detrimental to health and may be assessable via heart rate variability [20, 21]. Chronically low HRV is associated with impaired regulatory and homeostatic functions of the autonomic nervous system, which reduce the body’s ability to cope with internal and external stressors. Thus, HRV measurement is a noninvasive method that can be used to measure the autonomic nervous system in a variety of settings [22]. Studies show that for example in response to stress-inducing tasks, such as the Trier Social Stress Test, test participants show low parasympathetic activity, characterized by a decrease in High-Frequency Power (HF) and an increase in Low-Frequency Power (LF) HRV values [23–25]. The Standard Deviation of Normal-to-Normal heart beats (SDNN value) represents an index of physiological resilience to stress. When HRV is elevated and irregular, SDNN increases. On the other hand, especially when chronically stressed (e.g., at work), the SDNN value decreases [22, 26]. A low Root Mean Square of Successive Differences (RMSSD) value can also be an indicator of stress. Here, again, studies show that especially in chronic stress, values are lower than in non-stressed individuals [19, 25, 27]. However, it should be noted when evaluating HRV data that—beyond psychological stress—certain influencing variables must be considered. Age has a major influence on HRV. It increases initially, is highest in young adults, and decreases with increasing age [28, 29]. In addition, BMI correlates positively with sympathetic activity [30] and thus negatively with HRV [31, 32], whereas regular physical activity is associated with an increase in HRV [33, 34].

### Interventions to reduce stress

While health complaints are frequently observed, there are personal and organizational resources which can improve resilience towards occupational stress [35–37]. Personal resources with stress-protecting qualities include social support, coping style, self-efficacy, and optimism [38–41]. Pertaining to organizational resources, a recent systematic review identified supervisor support, job autonomy, and provision of work equipment to minimize stress [42].

Stress may differentially affect professions within the health sector [43]. For instance, nurses exhibit less health behaviors (e.g., physical activity) than physicians, pharmacists, and administrative health personnel [44]. According to Gerber & Pühse [45], physical activity may exert a stress-buffering effect and thus protect against physical and psychological illness. Also, within the nursing workforce, stressors vary between settings. For example, stressors in an outpatient care include long driving times and high emotional involvement with patients while this is less problematic in hospital settings [46].

Although a variety of stressors exist in the healthcare setting, evidence suggests that perceived stress can be reduced through participation in stress management interventions. For instance, mindfulness programs improve quality of life, anxiety, stress perception, and sleep quality [47, 48]. Physical activity-based studies showed improvements in autonomous nervous system function [49], accelerometric factors such as steps per day [50], BMI, sedentary behavior, MET, and physical activity levels [51]. More recently, mHealth interventions yield the potential to address stress in a low-cost, easy-to-implement fashion [52] with existing evidence for stress-reducing effects in different occupational settings [53].

Within the health care sector, efficacious stress reduction programs include Yoga and qigong [54], cognitive-behavioral interventions such as resilience training [55], mindfulness-based stress reduction (MBSR) [56], or multimodal combinations of aforementioned intervention types [57].

Despite the plethora of studies confirming the efficacy of stress reduction interventions, the evidence for health personnel is generally weak. Study rigor issues, for instance low total intervention time, small sample sizes, and failure to assess behavioral change undermine intervention quality [58]. Further, high risk of bias due to lack of both appropriate study designs and follow-up measurement points are common [54, 59].

The poor evidence base in the field of study is due to organizational, social, and individual reasons. According to Zhang et al. [60], participation in health promotion campaigns in health care facilities is often aggravated by various barriers. Specifically, amiss communication between management and staff, colleague peer pressure, insufficient staffing, top-down decision-making, and budget constraints can impede participation rates. Additionally, healthcare personnel are difficult to reach due to low motivation to change, low self-efficacy, and high psychological and physiological demands [61].

Moreover, due to differences in individual and organizational resources, stress management interventions should be tailored to the specific needs of participants. One possibility is to categorize subjects in terms of coping style when facing difficult work situations [60]. Further, there are individual preferences that need to be considered. For instance, health and other nonhealth-related outcomes (e.g., the value of a healthy future self and time costs, respectively) have differential impacts on the decision to engage in stress management [61]. Thus, one-size-fits-all interventions [62] should not be adapted for vulnerable populations as intervention success is limited [63].

In sum, to counteract stress effects in health personnel, low-cost, easy-to-implement, setting-specific, and need-tailored health promotion interventions are necessary. One way to address these issues is digital (mHealth) interventions.

### Digital Interventions for health promotion

Recent developments and studies highlight the opportunities of digital interventions to address the described concerns for implementing and evaluating interventions in the health care sector and the current stage of change readiness. Interestingly, internet-based interventions have been rarely implemented in the healthcare sector so far [64]. Digital health promotion programs can come in different forms: Web-based trainings (WBT) are presented on a secured online platform and assessed through an internet browser either on a smartphone or on a computer/laptop [65, 66], whereas app-based interventions come with a smartphone application only [67, 68]. However, there are also hybrid forms such as web apps.

Digital Interventions can be a low-threshold opportunity for health promotion and are a promising possibility to achieve prevention goals [69, 70], even though eHealth literacy is sometimes missing [65]. The free allocation of time and flexibility of availability were evaluated on a positive note. Combining such apps with so-called “wearables”, such as smartwatches or fitness trackers, could allow us to continuously record health data and thus constitutes various opportunities in the context of prevention work (Gamification, Just-in-time-adaptive interventions). By implementing “wearables” into digital health applications (apps), health-related data (e.g., sleep patterns, eating patterns, and exercise) could be recorded and interventions that meet individual needs could be derived based on this data [66]. Previous studies already found positive effects of stress apps on wellbeing. Harrer et al. [67] for example found that app-based stress management interventions improved stress, anxiety, and depression in college students. Another example stems from research by Economides et al. [68] who found that a mindfulness app intervention reduced stress and irritability, while it also increased positive affect. A systematic review and meta-analysis on web- and computer-based interventions for stress reduction illustrates international research efforts. Included studies have been carried out in Western countries (Austria, Switzerland, Germany, Great Britain, the Netherlands, Norway, Sweden, and the USA) and Japan with studies predominantly having been conducted in the USA. The meta-analysis further underlines that digital interventions have shown positive effects on stress outcomes in different samples in the countries mentioned [71].

At the same time, expectations towards health apps are high, 70% of health app users believe that these can strengthen self-motivation and 56% think that app use can improve health education [72].

In order to establish long-term health behavior change, a high level of adherence motivation during the intervention implementation is necessary, and therefore individually tailored approaches may be beneficial. Often, the adherence for digital health promotion programs is rather low which reduces their effectiveness [73]. Individual tailoring [74] or gamification could be approaches to address this problem. In one meta-analysis, researchers found that web-based tailored interventions clearly outplayed generic interventions with respect to health behavior change [75]. In particular, non-tailored interventions were found to decrease user satisfaction [76].

However, the definition of a tailored health app is unclear due to the lack of a framework for individualized app elements. In one of the few reviews that adequately addresses this issue, the authors enumerate the Individualized Elements in the app and grade whether it is a tailored or non-tailored mHealth intervention [77]. The evidence of this review is clear, however, that there is a wide range of potential approaches for individualization and that these are often accompanied by established behavior change mechanisms, yet the effectiveness of individual elements must first be investigated in stand-alone interventions, as tailored mHealth interventions are often multicomponent in nature.

One approach to design individually tailored digital solutions could be a focus on users’ personalities. A smartphone app that focuses on stress reduction thus firstly needs to focus on personality characteristics as studies showed that personality characteristics are associated with specific coping behavior [78], app usage behavior, and receptivity to gamification elements [79]. Focusing on personality characteristics also allows for app-tailoring. Additionally, it needs to address users on their current state of readiness for behavioral change App modules that focus on conflict solving skills and communication techniques could work to address those issues. To the best of our knowledge, to date there are no studies investigating the effect of tailored mHealth interventions to reduce stress in the healthcare sector.

In summary, for the development of a digital health intervention, the specific combination of different contents has to be considered. These are (1) evidence-based feasible interventions, (2) tailoring and individualization, and (3) additional elements to gain adherence and long-term usage. Therefore, the present study aims to compare both web-based vs. app-based and tailored vs. non-tailored stress management interventions. All included types of interventions were previously found to improve users’ wellbeing in different facets. While generalized web-based interventions that are designed for a broad user population require less technical effort than those containing individualization, a lack of individual tailoring appears to be a central issue when it comes to user motivation and willingness for behavior change. Individual-tailored app-based interventions on the other hand could address this need but require high technical effort as they rely on complex structures. One-size-fits-all interventions that are accompanied by an individual telephone coaching may be another option to allow a certain level of individual tailoring. Therefore, it is of high importance to compare the different approaches with regard to cost-benefit considerations.

With fitcor, we provide healthcare and administrative workers specific digital interventions. This protocol describes a randomized controlled trial (RCT) in which we investigate the effectiveness of the fitcor interventions. Therefore, our aim is to identify the most beneficial digital intervention with respect to the following goals: (a) reduce stress and associated consequences and/or symptoms, (b) increase self-management and self-efficacy, and (c) increase adherence.

The proposed trial is needed to address these aims for four reasons. It includes samples from three different health care settings (1), namely hospitals, stationary elderly care homes, and ambulatory care, which allows to draw conclusions on similarities and differences between the effectiveness of the interventions in the specific settings. It allows comparison of different digital interventions with regard to topic, complexity, or biofeedback inclusion (2). Additionally, it enables investigating stress both from an objective perspective facilitating physiological measures as well as from a subjective perspective facilitating self-report measures (3). Finally, we can derive conclusions on long-term effects of the applied intervention through our longitudinal approach [4]. We expect that each intervention will benefit participants as they get free access to usually pricey digital health improvement programs. But at the same time, participants will have to invest some of their time to conduct all three measurement time points. Other than that, no harms are assumed to appear directly caused by the intervention. Next to the effects on different outcome parameters, the whole approach will give us more information about the required composition of individualization and modularization in different occupational groups.

### Research questions

The main research question of this study is:

- □ Which digital intervention is most efficient for improving the stress management skills of health care and administrative personnel?

Regarding this main question, there might be some group-related differences to detect. Therefore, the following research questions should be answered:

- □ What are the differences in stress levels between nursing and administrative health personnel?
- □ What are the differences in sleep quality between nursing and administrative health personnel?
- □ How do intervention formats differ with regard to the effects on the processes of behavioral change?

Moreover, there are some outcome-related research questions:

- □ What is the association between the usage of mHealth intervention applications during working hours and stress levels of users?
- □ Is the effectiveness of the mHealth interventions determined by the age or gender of the participants?
- □ Is there a connection between stress and other outcomes such as back pain or physical activity levels?
- □ Do mHealth interventions contribute to a reduction in back pain?

Finally, the study should answer some acceptance and individualization related research questions:

- □ What is the rate of acceptance for the sensor-based mHealth interventions for nursing and administrative health personnel?
- □ What is the level of satisfaction for the sensor-based mHealth interventions for nursing and administrative health personnel?
- □ Do additional intervention requirements (e.g. for individualization) emerge as a result of the sensor screenings?
- □ What are the necessary requirements to ensure a long-term integration of sensor-based mHealth interventions to the daily routine of nursing and administrative health personnel?

## Methods

### Study design

The study is part of the project “Internet and app-based interventions to reduce stress in healthcare workers” (fitcor). The studies are conducted and described according to the Spirit checklist [80]. The study will be conducted as a longitudinal crossover design trial with five intervention groups and group comparisons (nurses vs administrative personnel). The study is part of the project “Internet and app-based interventions to reduce stress in healthcare workers” (fitcor). The studies are conducted and described according to the Spirit checklist [80]. The study will be conducted as a longitudinal crossover design trial with five intervention groups and group comparisons as described in Table 1 (nurses vs administrative personnel).

**Table 1:**
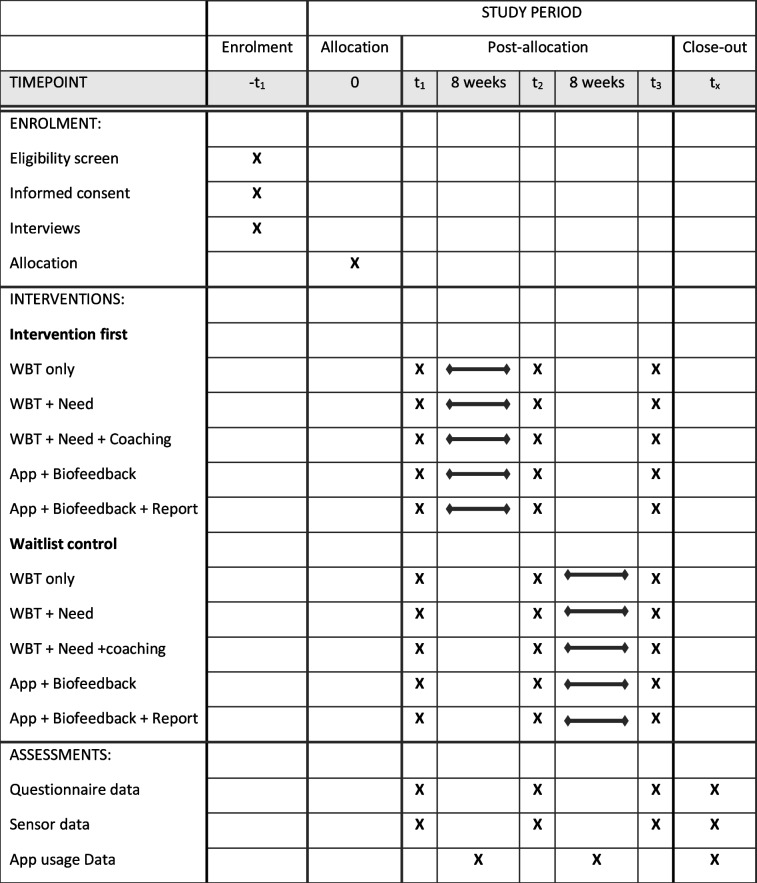
Schedule of enrollment, interventions, and assessments: Recommendations for Interventional Trials (SPIRIT) chart of the enrollments and assessments during randomized controlled trials

The five intervention groups are as follows:

1. Web-based digital stress management intervention (WBT only)
2. Web-based and need-oriented digital stress management intervention (WBT + Need)
3. Web-based and need-oriented digital stress management intervention with telephone coaching (WBT + Need + Coaching)
4. App-based stress management interventions with sensory biofeedback (App + Biofeedback)
5. App-based stress management intervention with sensory biofeedback and health report (App + Biofeedback + Report)

All participants of the intervention groups will receive a digital intervention. The waitlist control group will start the intervention after 8 weeks. Both, questionnaire and sensory data will be assessed:

1. At baseline (*T*_1_: pre-intervention/pre-waiting)
2. At 8 weeks (*T*_2_: post-intervention/post-waiting=pre-intervention)
3. At 16 weeks (*T*_3_: sustainability or post-intervention for waiting group; see Table 1).

### Participants

#### Eligibility and ethical approval

The crossover randomized controlled trial will include nursing staff and office workers aged 18 years or older from hospitals, stationary elderly care facilities, and ambulatory care providers. No clinical patients will be involved in the proposed study. Fluency in the German language as well as internet access via a smartphone device are prerequisites for study participation. All potential participants will be informed about the study and its procedure through a comprehensive informative video. The study is conducted in agreement with the principles of the Declaration of Helsinki and the guidelines of Good Clinical Practice. The recruitment of the participating nursing facilities involved cooperating health insurance companies, whereas the recruitment within the participating facilities will remain within the responsibility of the authors. Written informed consent will be obtained from all participants or their legal guardians before enrolment. Participants as well as their relatives or legal guardians can withdraw consent at any time. The local ethics committee of the TU Berlin, Germany, has approved the study protocol (No GR_14_20191217). The trial was registered at DRKS.de with registration number DRKS00021423 on 12 July 2021.

We used the program G*Power [81] to calculate the relevant sample size. To achieve the sample sizes required by power analysis with a β-error of 80% and an effect size of 0.25, we will need to include 700 participants. An effect size of 0.25 is considered a small effect, which is congruent with the literature on the effectiveness of eHealth/mHealth behavior change interventions [82, 83] and workplace health promotion interventions [84]. We expect a participation rate of 20% as this appeared to be a realistic participation rate in previous intervention studies in different small and middle-sized companies [85]. With an expected dropout rate of 20%, we will include additional 140 participants to ensure that the study results will still be eligible for data analyses.

#### Allocation and blinding

To prevent selection bias, the allocation of participants to the intervention groups and waiting control group will be assigned randomly by lot by the director of the study. There is going to be a random allocation at the individual level with the tool Research Randomizer using continuous block randomization. Sets of five numbers will be generated, representing the differing number of study groups. Each participant is then assigned the subsequent number on the block randomization list for group assignment. Therefore, each person can theoretically be assigned to any of the study arms. As participants will be assigned to an intervention group or the waiting control group by lot, no further mechanisms of implementing the allocation sequence is needed. To our best knowledge, there are no circumstances under which unblinding of the data assessors could be needed. Trial participants will be informed about which intervention group they are assigned to as they will need to receive the respective information to complete all necessary information and access the digital intervention programs. Outcome assessment will be blinded as the assessment is done in an online questionnaire that participants fill out independently. The sensor screening will also be done without including a third party as participants apply the sensor on their body by themselves. All data analyses will be run by blinded assessors.

#### Participant recruitment

Participants will be recruited in health care facilities in Germany. For this purpose, the health insurance companies contact the executives of their collaborating hospitals, stationary elderly care facilities, and ambulatory care providers. The executives will forward an explanatory video to their employees via in-house communication networks, whereupon they can voluntarily enter their contact details into an online tool to register for the study. Previous experiences of the researchers showed that approximately only half of all contacted nurses participated in intervention studies. Therefore, in order to reach the target sample size, we will contact double the participants than we will actually include in the study.

### Interventions

#### Explanation for the choice of comparators

In order to illustrate the advantages of mHealth interventions for the specific requirements of healthcare settings, office workers were chosen as a comparison group. The reason for choosing this comparison group is that office workers are often more likely to adapt to digital interventions due to their workplaces being better equipped in terms of technology. In addition, as described above, we developed five different study scenarios to be compared within the study. The reason for this selection is to reflect different levels of individualization in mHealth interventions across the different scenarios.

#### Intervention description

As described above, there are five different intervention scenarios, each including a WBT or an app, and each with different levels of individualization. This trial becomes particularly complex due to the need orientation of the WBT interventions. Depending on the needs of a person, he or she is assigned a different WBT. For example, a person who does not exercise enough and is very overweight is recommended a weight loss WBT, while a person who suffers from high stress levels is recommended a WBT with autogenic training or mindfulness. For this reason, a detailed list of the content covered in the respective apps or WBTs is provided in Table 2 below.

**Table 2:** Detailed list of the contents of the various needs-specific app and WBT modifications

Focus	Sub-focus	App				WBT							
		AusGleich	BIG Balance	myCare+	GetRest	Nutrition	Weight loss	Physical Activity	Spine Gymnastics	Mindfulness	Hatha Yoga	Sleep and Stress	Autogenic training
Individualization	Direct biofeedback	x	x	x	x								
	AVEM patterns	x											
	Telephone coaching					x	x	x	x	x	x	x	x
	Vocational setting	x	x	x	x								
	Need orientation					x	x	x	x	x	x	x	x
Stress and relaxation	Problem-focused	x	x	x	x								
	Deep breathing	x	x	x	x					x	x	x	x
	Mindfulness	x	x	x	x					x	x	x	
	Goal setting	x	x	x	x		x	x		x		x	x
	Gratitude journal	x	x	x	x					x	x	x	
	Positive psychology	x	x	x	x					x	x	x	x
	Autogenic training	x	x	x	x					x	x	x	x
	Progressive muscle relaxation	x	x	x	x					x	x		
	Body perception	x	x	x	x					x	x		
	Stress physiology												x
Sleep	Sleep habits	x	x	x	x								
	Sleep and shift work	x	x	x	x								
	Healthy sleep	x	x	x	x							x	
Physical activity	Stretching and yoga	x	x	x	x			x			x		
	Fascia						x	x	x				
	Behavior change	X	x	x	x								
	Activity habits	x	x	x	x								
	Endurance training	x	x	x	x			x					
	Anatomy						x	x	x				
	Spine health								x				

#### Strategies to improve adherence to interventions

In order to improve adherence to interventions, a user-centered approach was chosen to integrate experiences and test the functionality of the app internally and externally. Moreover, the information process about the study design implied an explanatory video, numerous flyers, and digital meetings. After agreeing to participate, numerous reminder emails were also created, which were automatically sent to the participants if they forgot to order the sensors or register.

#### Participant involvement

To ensure target group participation, the intervention conception was preceded by a qualitative needs assessment of the target group health care workers. We inquired about acceptance of the biofeedback system, the chest strap, and needs and requests for app content to improve need-tailoring and individualization aspects. For the sake of simplicity, we do not report the outcomes of this assessment in this study protocol.

### Outcome measures

The assessment will apply a selection of standardized questionnaire measures (cited below) as well as sensor-based physiological and vital parameter measures (measured by Corvolution CM300, which includes ECG circuit, 3-axis acceleration and rotation rate chip, air pressure chip, thoracic impedance chip, and temperature chip). Additionally, demographic characteristics, such as age, gender, and job hierarchy, will be assessed.

#### Primary outcomes

All primary outcomes will be assessed at baseline (*T*_1_), at follow-up measurement after 8 weeks (*T*_2_), and at follow-up measurement after 16 weeks (see Table 1).

##### Stress and relaxation

While perceived stress and stress symptoms will be assessed in a questionnaire format, heart rate variability (HRV) and sympathovagal balance are captured by the sensor.

- □ *HRV*: HRV measurement will be used to determine the ability of the heart to respond to daily physiological and psychological stimuli. The following HRV parameters will be used for the assessment of psychological health and stress: SDNN (ms), RMSSD (ms), LF (ms2), HF (ms2), and the LF / HF-Ratio.
- □ *Perceived Stress*: Stress perception will be assessed via the PSS-4 questionnaire. The PSS-4 is the short version of the original PSS-10, developed by Cohen et al. [86], which measures the extent to which respondents perceive their life situation as unpredictable, uncontrollable, and overloaded, and thus feel stressed. The 4-item version incorporates the statements: “In the last month how often have you felt you were unable to control the important things in your life?,” “In the last month how often have you felt confident about your ability to handle your personal problems?,” “In the last month how often have you felt that things were going your way?,” “In the last month how often have you felt difficulties were piling up so high that you could not overcome them?”. The 5-point response scale ranges from 0 = never to 4 = very often. Internal consistency is good at *α* = 0.77 [87].
- □ *Subjective stress symptoms*: A self-developed questionnaire asks respondents to rate 10 stress symptoms (e.g., I often feel alone, abandoned, and isolated) on a 5-point Likert scale (not true—true).

##### Sleep quality

Sleep quality will be measured via both sensor data (sleep duration and sleep recovery) and validated questionnaires (subjective sleep quality, sleep apnea risk).

- □ *Sleep duration*: Determination of sleep duration follows the method of Cole et al. [88]. Classification of sleep duration occurs as hours/day, where <6 h/day = insufficient, 6–9 h/day = sufficient, and >9 = excessive [89].
- □ *Sleep recovery*: ECG parasympathetic activation will measure sleep recovery. Via the sensor, participants’ recovery will be classified as poor, moderate, or good.
- □ *Subjective sleep quality*: The Pittsburgh Sleep Quality Index (PSQI) will be used to collect subjective sleep quality data. The index is composed of 18 items covering seven relevant areas (subjective sleep quality, sleep latency, sleep duration, habitual sleep efficiency, sleep disturbances, use of sleep medications, and dysfunction during the day within the past four weeks) [90]. An example item is “During the past 4 weeks, how often have you slept poorly because you woke up in the middle of bedtime or much too early?” Across the seven areas, a total of 0–21 points can be achieved. Scores above 11 reflect poor sleep quality, whereas a score of 5 and below reflects good sleep quality. The German version demonstrated good internal consistency (*α* = 0.75 [91];).
- □ *Sleep apnea risk*: The snoring, tiredness during daytime, observed apnea, and high blood pressure with BMI, age, neck circumference, and gender (STOP-Bang) questionnaire will be used to identify obstructive sleep apnea (OSA) risk. The STOP-Bang questionnaire asks four yes/no questions (“Do you snore loudly (louder than talking or loud enough to be heard through closed doors)?”, “Do you often feel tired, fatigued, or sleepy during daytime?”, “Has anyone observed you stop breathing during your sleep?”, “Do you have or are you being treated for high blood pressure?”) to quickly screen OSA risk. Respondents are assigned a score for each item (no = 0, yes = 1) for a possible score of 0 to 8 points. Using a cut-off score ≥ 3, the STOP-Bang questionnaire exhibits high sensitivity to detect any OSA (84%), moderate-to-severe OSA (93%), and severe OSA (100%) [92].

##### Physical activity and fitness

Sensor-based measures of physical activity and fitness measures include daily steps, energy expenditure, activity intensity and variety, physical inactivity, and BMI.

- □ *Steps*: Accelerometer-based measurement of steps (steps/day) will be recorded, and participants will be categorized as low active (<5000 steps/day), moderately active (5000–10,000 steps/day), and highly active (>10,000 steps/day) adapted from the Tudor-Locke & Bassett [93] framework (originally: sedentary (<5,000 steps/day), low active (5000 to 7499 steps/day), somewhat active (7500 to 9999 steps/day), active (10,000 to 12,499 steps/day), and highly active (>12,500 steps/day)).
- □ *Total daily energy expenditure*: Based on the work of Livesey [94], daily activity will be computed as the discrepancy from the individual basal metabolic rate (BMR; MJ/day) requirements determined by age, sex, and body weight (kilograms). Participants with total daily energy expenditure <1.5, BMR are considered low active, 1.5–1.7 BMR reflects moderate daily activity, and daily energy expenditure >1.7 BMR will be treated as highly active.
- □ *Activity variety*: Metabolic equivalent of task (MET) minutes/day will be assessed for daily activities [95].
- □ *Activity intensity*: Activity intensity will be computed as mean heart rate in relation to minimum and maximum rate during the measurement period.
- □ *Physical inactivity*: Physical inactivity will be assessed as the number of minutes per days those participants exhibit waking inactivity (sitting, standing) Classification is as follows: <60 min/day = good, 60–240 min/day = moderate, >240 min/day = poor. Also, inactivity disruptions will be measured, operationalized as a disruption of a ≥ 30-min inactivity period for at least 1 min (e.g., short walk after a 30-min sitting period).
- □ *BMI*: The BMI will be computed (kg/m^2^). According to the WHO [96], participants will be classified as underweight (<18.5 kg/m^2^), normal weight (18.5 to 25 kg/m^2^), and overweight (>25 kg/m^2^). Height and weight are assessed by questionnaire.

#### Secondary outcomes

##### Behavioral and experiential outcomes

In addition to the three primary outcome domains of stress, sleep, and physical activity, information was collected on experienced back pain, health behaviors, work-related behavior and experience patterns, and personality. These were used to tailor the app. All behavioral and experiential secondary outcomes will be assessed at baseline (*T*_1_), at follow-up measurement after 8 weeks (*T*_2_), and at follow-up measurement after 16 weeks (see Table 1).

- □ *Back pain*: The back complaints of the last 7 days are surveyed using three self-developed items, addressing the location and situations in which back complaints occur.
- □ *Health behavior*: Based on the Health Action Process Approach, 22 items were adapted from Schwarzer [97] to capture motivational and volitional mechanisms that influence individual stress management practices. Items include statements regarding self-efficacy, intentions, outcome expectancies, planning, and actual stress coping behavior (e.g., “How certain are you that you can execute the exercise in the app?”). All items are assessed on a 5-point Likert scale (e.g., 1: not at all certain–5: very certain).
- □ *Work-related behavioral and experiential patterns*: To assess the Work-related Behavior and Experience Patterns (German acronym AVEM), we included four items that were self-developed based on the 44-item original version by Schaarschmidt & Fischer [98]. The original questionnaire comprises 44 questions covering eleven dimensions (subjective meaning of work, occupational ambition, energy expenditure readiness, perfectionism, emotional distancing ability, resignation tendency, offensive problem coping, internal peace and balance, perceived work success, life satisfaction, and perceived social support). Respondents are clustered into one of four patterns (health, conserving, overexerting, and burnout) that can be used to identify individual intervention targets. The condensed 4-item version used for this study was validated in an online survey (e.g., “My job is important to me, but I also manage to distance myself from my work and thus maintain a high quality of life.”). The internal consistency of the original version ranges from 0.79 to 0.87 [99].
- □ *Personality*: We will include the Big-Five-Inventory-10 (BFI-10) comprising ten items that economically measures personality based on the five-factor model [100]. The procedure was shown to be reliable in evaluation studies with a retest reliability of 0.49 to 0.84 and exhibited good content as well as convergent validity. An example item of this scale is “I trust others easily, believe in the good in people.”

##### App-related outcomes

During the intervention, usage data was also collected that included information about satisfaction with the app, modules completed, intensity of use, and duration of use. App-related secondary outcomes will only be assessed during the intervention period (see Table 1).

- □ *Satisfaction with internet-based programs*: To assess the satisfaction with internet-based training, we include the Client Satisfaction Questionnaire-Internet (CSQ-I), which measures the acceptance towards digital interventions with eight items (e.g., “I am satisfied with the amount of help I received from the training”). The questionnaire has very good reliability with *α* = 0.94 [101].
- □ *App usage data*: Tracking of intervention module finalization and total time spent with allocated modules will be performed.

### Data collection, management, and analysis

Relevant outcome variables will be assessed at three time points. The different measurement points are the same for all five intervention groups. The baseline measurement (*T*_1_) is first carried out on the participants directly prior to beginning with the interventions. The post-intervention measure will be performed immediately after the interventions are completed (*T*_2_). After another 6 weeks of no intervention, the sustainability measurement (*T*_3_) will be conducted. For the waiting control group, the first measurement (*T*_1_) will be followed by a period of eight weeks of waiting. The second measurement (*T*_2_) will be conducted after this waiting period. Then participants of the waiting control group will receive one of the interventions and then have their third measurement directly after finishing the respective intervention (*T*_3_). At all three measurement points, the same variables, namely physiological stress, perceived stress, satisfaction with the digital intervention, personality, work-related behavior patterns, and team conflict will be measured.

The respective measurement tools that will be applied are described in the outcomes section including information on reliability and validity. In order to promote data quality, we include evaluated scales that appeared to be reliable and valid in previous studies. We will not conduct duplicate measurements. The collected data will be processed pseudonymously in digital form (from the initial measurement to the sustainability study—approx. 12 weeks).

In order to ensure pseudonymization and simultaneously ensure subsequent deletion of the data, each participant creates a five-digit individual code word immediately after signing the consent form when answering the initial survey, consisting of the first letter of their mother’s first name, the first letter of their favorite color, the first letter of their place of birth, the last digit of their year of birth, and the last digit of their day of birth (e.g., MGF01). This code is used instead of other identifier in all subsequent measurements. There is a coding list on paper that links the name to the code but is only accessible to the investigators and the project manager. The coding list is kept in a lockable cabinet or safe and is destroyed after the data collection is completed. If a respondent wishes to delete their data retrospectively, they can use the five questions mentioned above to reconstruct their code word and thus request deletion of the data. After completion of the sustainability measurement, the code used to pseudonymize the employees will also be replaced by assigning a combination of numbers (e.g., 1647) and thus anonymized. Non-anonymized data sets are deleted by the university and the cooperation partners involved in the data collection after completion of the data collection. Data will only be processed in anonymized form within the framework of the study and for subsequent scientific use as well as for publication of the study results.

The anonymized data sets will be stored on a password-protected project folder of the TUB cloud of the TU Berlin for the duration of the project until the completion of all scientific work and associated data analyses. Subsequently, the data will be transferred to a research data repository of the TU Berlin and stored there for a period of 10 years. Access to the server is granted by assigning passwords to the external project partners. The rights to assign the passwords lie with the project leader PD Dr. Bettina Wollesen. After completion of the project, all raw data collected can be made publicly available in anonymized form in a research data repository for an unlimited period of time as part of the Open Science efforts of the scientific community and in accordance with the Berlin Declaration. Personal information about the participants will be protected by pseudonymization of the data sets described above.

#### Statistical analysis

Primary and secondary outcomes will be analyzed using SPSS software. The study will allow a comparison between all summarized intervention groups with the waiting control group, as well as a comparison between the different intervention groups. Additionally, participation rates will be included in the analyses. Sample characteristics will be explored applying descriptive statistics. Standard analyses adjustments will be made to adjust for baseline differences between groups, in case there are any. For missing data, sensitivity analyses will be conducted to compare results with the complete case analysis. Further, different options for imputation will be considered. Differences between intervention groups and the control group will be investigated using *χ* ^2^ tests for categorical variables and independent sample *t*-tests for continuous variables. General linear mixed models will be applied for the statistical analyses of primary and secondary outcomes. Group-based trajectory modelling will also be applied if feasible given the data situation. The models will be adjusted by baseline value and potential confounders, such as staff field of working (hospital, stationary elderly care, ambulatory care) or age. *P* values <.05 will be considered as statistically significant and effect sizes of >.3 will be regarded as clinically significant. If appropriate, 95% CI will be reported with the *p* values as well. If feasible, missing data will be imputed through multiple imputation.

#### Monitoring

The research team will promote participant retention through close personal support. Contact persons will always be available for any occurring questions or problems. Further regular reminder emails will be sent out as soon as participants appear to fade out of completing the interventions. A respective data monitor will be named and be responsible to supervise the active participation. If there is no response to the reminder emails, participants will be contacted by phone by the research team. If participants decide to withdraw from the intervention, no further data of them will be collected.

All researchers involved in the study will monitor the data and report in case of appearing inconsistencies or other problems. In the beginning of the project, data monitoring will take place on a daily basis. As soon as the study runs smoothly, data will be monitored on a weekly basis.

We do not expect that any adverse effects appear during the study. However, in case any solicited, and spontaneously adverse events are reported, the research team will inform the supervisor and decide based on her expertise how to deal with the events. The investigators will audit the trial conduct on a weekly basis to inspect how the trial is going and whether any adaption or intervention is necessary. This will be done in collaboration with the trial sponsor. In case any protocol amendments will be necessary, the ethical committee that granted approval for the present trial will be informed immediately. Additionally, all participants will be informed about changes. The results of the trial will be reported to participants and their institutions in an anonymized report that is specifically designed to be understandable by people without a scientific background. The study results will further be made available to the public and interested researchers through an open access publication in peer-reviewed journal.

## Discussion

The development of a digital intervention to improve the individual’s abilities for stress management in different working settings, especially in the health sector, is facing several requirements. Next to the integration of evidence-based feasible interventions, these contents need to be tailored and individualized to gain adherence and long-term usage. Therefore, the main aim of the study is to compare web-based and app-based stress management interventions to identify the most beneficial digital intervention to (a) reduce stress and associated consequences and/or symptoms, (b) increase self-management and self-efficacy, and (c) increase adherence.

Regarding the overall aim, previous studies have shown that the nursing occupation is strongly linked to stress experiences [9]. The working conditions within the health care sector are accompanied by staff shortage and poor health outcomes, which were exacerbated by increased workloads during the pandemic [102]. Previous studies have shown chronic stress and resulting physical, cognitive, and emotional strains in nursing personnel [103, 104]. Regarding the physical conditions, chronic stress leads to reduced heart rate variability [21].

As administrative personnel do not have the same level of intimate encounters with patients, they are less likely to experience work stress. Also, shift work is an independent stressor, which is less of a problem for administrative personnel. Therefore, we suppose the two target groups within our study might show differences in their chronic stress conditions and referring physical reactions (expressed by heart rate). Moreover, it has been shown that the physical conditions might also lead to reduced sleep quality or duration [105]. With respect to the different nature of work-related burdens within nursing and administrative personnel, these circumstances might also lead to differences in individual sleep quality.

Due to previous studies providing positive effects of app-based stress management and mindfulness interventions on wellbeing, reduced stress, anxiety, and depression [67, 68], these types of interventions should be tailored and adopted to specific requirements of the target groups within this study. Also, work-specific stressors and back pain are known to be interrelated, with a potential bidirectional causality. For both groups, the underlying mechanisms might be different. While nursing personnel has to compensate heavy loads on the spine according to care processes with awkward body positions under time pressure, administrative personnel have long sitting periods without moving [103, 106]. However, despite the different strains on the musculoskeletal system, stress is commonly associated with increased blood pressure and an augmented perception of pain [20, 107].

Overall, the study results will help to gain more evidence of the effectiveness of these interventions for health personnel according to positive benefits on stress reduction. This will be gained by an appropriate small sample size, appropriate study designs, and follow-up measurements to address the relevant aspects identified [54, 59]. Moreover, we expect to find a positive correlational link between stress level of participants and back pain severity as well as a negative correlational link between stress level of participants and physical activity level [11].

Also, the digital interventions allow more temporal flexibility for usage and therefore might help to overcome organizational, social, and individual reasons and barriers for participation proposed by Zhang et al. [108]. Additionally, healthcare personnel are difficult to reach due to low motivation to change, low self-efficacy, and high psychological and physiological demands [109]. Increasing the motivation and improving the stress coping related self-efficacy of healthcare personnel can be achieved by including measures of behavior change mechanisms. Whether a health intervention will be successful in changing a behavior in the desired direction is contingent on changes in motivational and volitional aspects, such as intention to change and planning behavior. As individualized interventions are theorized to be more motivating than generalized interventions, we expect the need of tailored conditions to be associated with improved self-efficacy, intentions, planning, and actual behavior compared to generic and waitlist control conditions. These changes will be accompanied by health behavior stages (i.e., from non-intending to intending to acting). At the same time, organizational barriers (e.g., inadequate social support, high job demands) for behavioral change are known to prevail in the nursing sector compared to facilitators [110] which influence the change outcomes. Organizational barriers may be less problematic in the administrative setting, which could show in better intervention results for administrative vs nursing personnel. The individualization and the just-in-time-adaptive intervention [111] approach of the sensor-based app interventions are expected to be particularly successful in decreasing perceived work stress. Web-based, non-individualized interventions will likely also yield desirable results, however to a lesser degree.

One-size-fits-all interventions have been found to produce fewer desirable results than need-tailored interventions [63]. We expect biofeedback-based, need-tailored digital interventions to be superior in terms of stress reduction and physical activity improvements than generalized, nonspecific online courses. However, the presented biofeedback system has not been tested before systematically. It might therefore be the case that additional intervention necessities emerge by means of the sensor screenings. As an example, if sleep recovery is found to be of particular low quality, recommendations will be made to specifically target sleep in future interventions.

Next to the positive effects of tailoring the interventions to gain higher effects of individual stress responses, individualization might be expected to increase the adherence of the participants [112]. A continuous participation in an intervention program is necessary to gain positive adaption and long-term effects. The interventions of this study integrate different forms of strategies (e.g., tailoring, self-tracking) that are helpful to maintain a certain behavior [113, 114]. To our knowledge, there is no study that compared these different approaches within care settings, yet. Therefore, with this study, we detect new insights into the most beneficial composition of the program with respect to adherence according to the acceptance rates.

Further, moderators for intervention effects will be analyzed. For instance, a higher degree of usability for younger participants vs elderly participants may be apparent. However, it has recently been shown that elderly people are improving their e- and mHealth literacy [115]. Thus, whether or not relatively young participants profit more from the current intervention will be analyzed.

In summary, this trial integrated evidence-based contents demonstrating positive effects on stress management and converted these contents for the use of digital health in the context of healthcare work. Moreover, the approach integrates individualization to the digital offers to improve effectiveness and adherence.

### Limitations

This study comes with limitations: The participants will be asked to fill in questionnaires that come with known issues such as social desirability, tendency to the middle, common method bias, and subjectivity due to self-perception. Further, a lack of time in a highly stressed population that nurses are may lead to answering under time pressure and thus not reading the questions of the survey with necessary attention. Another possible limiting factor can be the interchange about the health promotion programs between nurses in different groups which might cause changes in the waiting control group. Further, work culture, team environment, and management can affect intervention uptake and thus the interventions effectiveness.

## Practical implications

The current study can provide useful information for workplace health promotion interventions aimed to improve work ability of health care workers. This large-scale trial is the first to assess the feasibility of an mHealth intervention for a highly stressed target group that requires special societal attention as nursing shortages are present in many countries. To counter health care worker disease and job turnover, health promotion experts should consider both the positive preliminary results and issues pertaining to adherence and participant attrition.

## Trial status

By the time of submission of this study protocol, we have already received a positive ethics vote from the local ethics committee of the Technical University of Berlin, as well as successfully registered the study with the DRKS (German Registry for Clinical Trials; available on https://drks.de/search/de/trial/DRKS00024605). We are currently in the participant recruitment phase. At the time of the initial study protocol submission, we entered the recruitment phase. We began recruiting in April 2021 and completed in March 2022. In parallel to the recruitment phase, the first facilities initiated the measurement phase. To date, data measurement has started in September 2021.

## Data Availability

All participant information and data will be stored securely and identified by a coded ID number only to maintain participants’ confidentiality. It is planned to transfer the data to an open access repository. The datasets analyzed during the current study and statistical code are available from the corresponding author on reasonable request, as is the full protocol.
